# A divergent and stereoselective synthetic strategy for tetraarylethylene-based AIEgens

**DOI:** 10.1093/nsr/nwab015

**Published:** 2021-02-12

**Authors:** Zheng Zhao, Ben Zhong Tang

**Affiliations:** School of Chemistry and Chemical Engineering, Southeast University, China; Department of Chemistry, The Hong Kong University of Science and Technology, China; Department of Chemistry, The Hong Kong University of Science and Technology, China; HKUST-Shenzhen Research Institute, China; Center for Aggregation-Induced Emission, State Key Laboratory of Luminescent Materials and Devices, South China University of Technology, China

**Figure 1.**
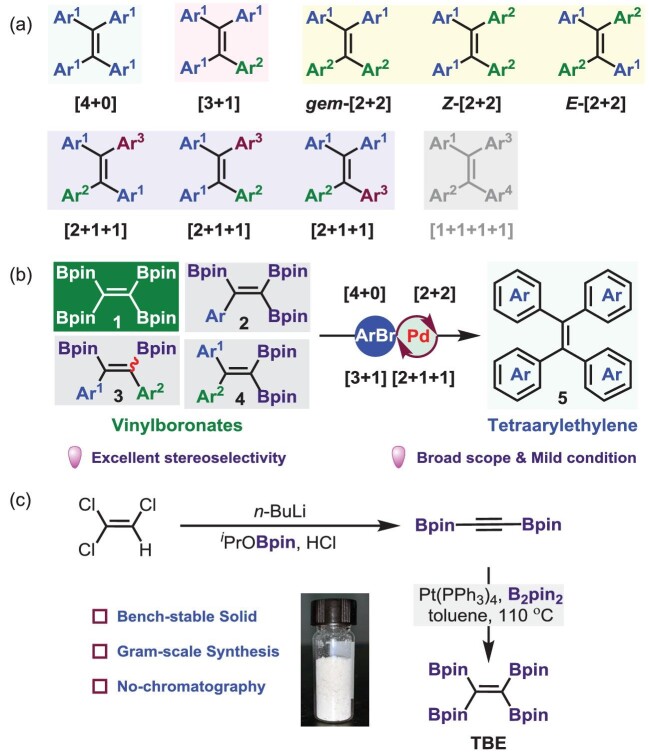
**(**a) Classification of tetraarylethylenes (TAEs). (b) Synthesis of TAEs (**5**) from vinylboronates (**1**–**4**). (c) Preparation of tetraborylethylene (TBE, **1**). Adapted from ref. [5] with permission.

Since the coinage of the concept of aggregation-induced emission (AIE) in 2001 [1], AIE molecules have been widely utilized as biological probes, chemical sensors, and optoelectronic and stimuli-response materials [2]. Tetraarylethylenes (TAEs) are archetype luminogens with an AIE attribute (AIEgens) and have been used as frameworks for further functionalizations, structure-property relationship studies and application explorations [3]. According to the aryl substituents, TAEs can be divided into five groups, i.e. [4+0], [3+1], [2+2], [2+1+1] and [1+1+1+1] (Fig. 1a). The structural diversity of TAEs offers a powerful platform and great opportunities for elucidating differences in properties and functions of the stereoisomers in molecular and aggregate levels. While traditional methods such as McMurry reactions and Rathore’s procedures are effective for TAE synthesis, the stereoselectivity of these protocols is a daunting problem. Furthermore, due to the structural similarity, the truly flexible and stereodefined synthesis of TAE isomers represents a daunting challenge for researchers in the area of AIE study. For example, common access to pure *E*-[2+2]- and *Z*-[2+2]-TAEs relies on high performance liquid chromatography (HPLC) separation of their *E/Z* mixtures or the introduction of polar functional groups into the TAE skeleton to enlarge the difference between *Z* and *E* isomers in shape and polarity [4].

Very recently, Zhao *et al*. developed a general and effective synthetic approach to structurally diverse and stereodefined TAEs from vinylboronates via Suzuki-Miyaura cross-coupling (Fig. 1b) [5]. Significantly, a new tetraborylethylene (TBE, **1**) was developed for the synthesis of [4+0]-TAEs from commercially available trichloroethylene. It is worth pointing out that TBE is air-stable and could be amenable for gram-scale synthesis without chromatography purification (Fig. 1c).

Through variation and assessment of reaction parameters, the optimal conditions for the syntheses of [4+0]-TAEs from TBE via four-fold coupling were identified. Subsequently, under the optimalized reaction conditions, the stereoselective syntheses of [4+0]-, [3+1]-, [2+2]- and [2+1+1]-TAEs from the corresponding vinylboronates and aryl halides were established, manifesting broad substrate scopes and excellent functional group tolerance. Notably, a variety of pure *E-*[2+1+1]-TAEs were prepared by stepwise Suzuki-Miyaura cross-coupling of triborylalkenes, representing an important complement to the existing methods that suffer from separation difficulty. The stereochemistry of *E-*[2+1+1]-TAEs was unambiguously assigned by X-ray crystallographic analysis of the representative products. Additionally, the robustness and synthetic utility of this protocol were demonstrated by the construction of TPE-cored crown ether and organoplatinum(II) metallacycles.

In summary, Zhao *et al*. have successfully developed TBE and demonstrated its utilities in the syntheses of [4+0]-TAEs. The divergent and stereoselective syntheses of [3+1]-, [2+2]- and [2+1+1]-TAEs via multiple couplings of vinylboronates with aryl bromides were also illustrated. These coupling reactions feature a broad substrate scope and excellent functional group compatibility due to the mild reaction conditions. Inherent from the unique stereoselectivity, it is envisioned that this protocol will be conducive to the straightforward preparations of TAEs with desired geometry and the exploration of efficient AIE-active functional TAE materials with specific applications.

***Conflict of interest statement*.** None declared.
